# Copper homeostasis and organelle-dependent mechanisms in bone metabolism: implications for osteoporosis therapy

**DOI:** 10.3389/fcell.2026.1880910

**Published:** 2026-08-10

**Authors:** Jialun Jiang, Jiawen Lin, Ziyu Zhou, Hongwei Pan, Pengju Zhao, Dandan Ma

**Affiliations:** Department of Stomatology, Zhejiang Chinese Medical University, Hangzhou, China

**Keywords:** bone metabolism, copper, cuproptosis, organelle, osteoporosis

## Abstract

**Background:**

Osteoporosis is a prevalent metabolic bone disorder driven by an imbalance between osteoblast-mediated bone formation and osteoclast-mediated bone resorption, and current pharmacological options remain limited by adverse effects and incomplete mechanistic targeting. Copper, an essential trace element, has long been linked to bone mineral density through population-level dietary surveys, but whether and how copper mechanistically shapes osteoblast and osteoclast function at the subcellular level has not been systematically reviewed.

**Methods:**

We synthesised genetic, clinical, and cell-biological evidence—including copper-transporter mutation models (Menkes and Wilson disease), organelle-specific trafficking studies, and mechanistic work on copper-dependent enzymes and chaperones—to construct an organelle-resolved account of copper handling in bone cells, extending beyond the mitochondria and trans-Golgi network to include the endoplasmic reticulum and the lysosomal/endosomal system.

**Results:**

Copper regulates bone metabolism through distinct, organelle-specific, and cell-type-specific mechanisms. In mitochondria, physiological copper supports oxidative phosphorylation and osteogenesis, whereas copper overload can trigger cuproptosis, a mechanistically distinct form of cell death mediated by FDX1-dependent aggregation of lipoylated tricarboxylic acid cycle proteins; whether this pathway selectively affects osteoclasts remains an open, and in part contested, question, since the hypoxic bone marrow niche may confer glycolysis-associated protection. In the trans-Golgi network, ATP7A/ATP7B-dependent maturation of lysyl oxidase supports collagen cross-linking, while COMMD1 restrains NF-κB-driven osteoclastogenesis. Emerging evidence implicates copper-dependent PERK activity in endoplasmic reticulum proteostasis relevant to osteoblast secretory function, and lysosomal CTR2-mediated copper release as a candidate regulator of mTORC1-autophagy signalling in osteoclasts, although direct evidence in bone cells for both pathways is still lacking.

**Conclusion:**

Copper acts as a compartment-specific and cell-type-specific regulator of bone remodelling rather than a uniform, dose-dependent factor. Trans-Golgi-network-targeted strategies to enhance lysyl oxidase maturation are the most mechanistically mature translational direction, whereas mitochondria- and lysosome-targeted approaches remain hypothesis-generating and require direct validation in osteoblasts and osteoclasts before therapeutic development can reasonably proceed.

## 1 Introduction

Osteoporotic fractures pose a major threat to health, particularly in older adults, and impose a substantial socioeconomic burden (Wu et al., 2021). Bone remodelling depends on the balance between osteoblast-mediated bone formation and osteoclast-mediated bone resorption. Any factor that decreases osteoblast activity or increases osteoclast activity shifts this balance toward excessive bone resorption, leading to deterioration of bone microarchitecture, reduced bone strength, and increased susceptibility to fragility fractures. Current anti-resorptive therapies, including estrogen, bisphosphonates, denosumab, and calcitonin, have been used clinically for many years, but their long-term use can be limited by adverse effects. Anabolic agents, such as parathyroid hormone analogues, also have clinical limitations, including nausea, dizziness, and muscle cramps. Therefore, identifying new mechanisms and therapeutic targets remains important for improving osteoporosis (OP) treatment.

The dynamic balance of bone metabolism depends on the synergistic regulation of osteoblasts and osteoclasts, and the metal ion microenvironment plays a key role in this process. Recent studies have revealed that copper, as an essential trace element, not only participates in mitochondrial energy metabolism and antioxidant defense, but also affects bone homeostasis through the regulation of other cellular organelles. Its deficiency may lead to bone metabolism disorders and contribute to OP development. Epidemiological studies have linked dietary and total copper intake to increased bone mineral density (BMD) and reduced osteoporosis (OP) risk in adults (Fan et al., 2022). However, cross-sectional studies cannot establish a causal relationship between the two, nor can they elucidate the underlying mechanisms. Therefore, we sought evidence of an association in genetic, clinical, and pharmacological intervention studies.

Genetic, clinical, and pharmacological evidence further supports a causal relationship between copper dyshomeostasis and bone dysfunction. In mammals, ATP7A and ATP7B are the major copper-exporting ATPases that regulate intracellular copper distribution, particularly through the trans-Golgi network (TGN). Patient-derived induced pluripotent stem cells (iPSCs) from Menkes disease (MD), a disorder caused by loss-of-function mutations in ATP7A, show defective mesenchymal stem cell (MSC) differentiation, reduced alkaline phosphatase activity, impaired matrix mineralisation, and downregulated osteogenic gene expression. ATP7A knockdown in wild-type MSCs recapitulates these osteogenic defects and is accompanied by reduced lysyl oxidase (LOX) activity (Kim et al., 2015). At the patient level, radiographic imaging of genetically confirmed MD has revealed wormian bones, rib flaring, metaphyseal spurring, and periosteal reactions, consistent with impaired collagen and elastin cross-linking secondary to ATP7A dysfunction (Zhu et al., 2024). Conversely, Wilson disease (WD), caused by ATP7B dysfunction and chronic copper overload, is associated with low bone mass and fragility fractures; in a cohort of 85 adults with WD, 51% had a history of fracture (Quemeneur et al., 2014), and a recent large cohort study identified age, sex, and skeletal symptoms as risk factors for osteoporosis in WD (Zhu et al., 2025). Pharmacological evidence also supports a functional role for copper in bone remodelling, as copper chelation with tetrathiomolybdate suppresses RANKL-induced osteoclastogenesis and LOX-dependent bone resorption *in vivo* (Morisawa et al., 2018). Together, these findings establish copper as a modulator of bone cell function and justify a mechanistic, organelle-level analysis of copper regulation in osteoblasts and osteoclasts.

Animal models provide complementary systems for dissecting ATP7A- and ATP7B-dependent copper trafficking *in vivo*. Atp7a-mutant “mottled” mice phenocopy key features of human MD, including connective tissue and skeletal abnormalities, and remain a major model for studying ATP7A-dependent copper transport (Lenartowicz et al., 2015). By contrast, Atp7b-deficient models, including toxic-milk mice and Atp7b knockout mice, reproduce the hepatic copper accumulation characteristic of WD and provide tools for investigating the systemic consequences of chronic copper overload. Although these models have been used extensively to study hepatic and neurological phenotypes, their skeletal phenotypes remain less well characterized, representing an important gap in understanding copper-related bone disease.

Thus, these genetic, clinical, and interventional data move the copper–bone relationship beyond correlation and justify a mechanistic, organelle-level dissection of how copper regulates osteoblast and osteoclast function.

## 2 Copper homeostasis

### 2.1 Organelle-level copper regulation in osteoblasts and osteoclasts

To contextualise the compartment-by-compartment analysis that follows, we first outline an integrative framework of copper trafficking in bone cells and its distinct wiring in the two principal cell types of bone remodelling—osteoblasts and osteoclasts.

Copper entering through the high-affinity copper transporter 1 (CTR1) is captured by a small set of chaperones—ATOX1, CCS, and COX17—which route it to three major destinations: mitochondria, where COX17 loads copper onto cytochrome C oxidase (COX) for oxidative phosphorylation and where copper overload triggers cuproptosis; the trans-Golgi network (TGN), where ATOX1 delivers copper to ATP7A/ATP7B for the maturation of secreted cuproenzymes such as lysyl oxidase (LOX); and the nucleus, where LOX-derived signals and ATOX1 itself modulate RANKL-and NFATc1-dependent transcriptional programmes. Two additional compartments—the endoplasmic reticulum (ER) and the lysosomal/endosomal system—are increasingly recognised as integral nodes of this network. In osteoblasts, the massive flux of type I collagen and LOX through the secretory pathway intimately couples ER proteostasis to copper-dependent enzyme maturation. The lysosomal/endosomal system serves as a copper storage and recycling reservoir (via CTR2) and, in osteoclasts, also supplies the acidification and degradative machinery essential for bone resorption.

Crucially, copper trafficking and organelle function form a bidirectional relationship. Organelle dysfunction actively reshapes copper handling: ER stress activates the UPR kinase PERK, whose kinase activity is itself copper-dependent (Bond Newton et al., 2025), creating a feed-forward loop; lysosomal impairment dampens CTR2-mediated copper release, secondarily starving mitochondrial and TGN pathways of copper (van den Berghe et al., 2007). Bone cell fate is therefore best viewed not as a linear cascade but as a dynamic, cross-organelle signalling system in which copper flux and organelle proteostasis mutually constrain one another.

**FIGURE 1:**
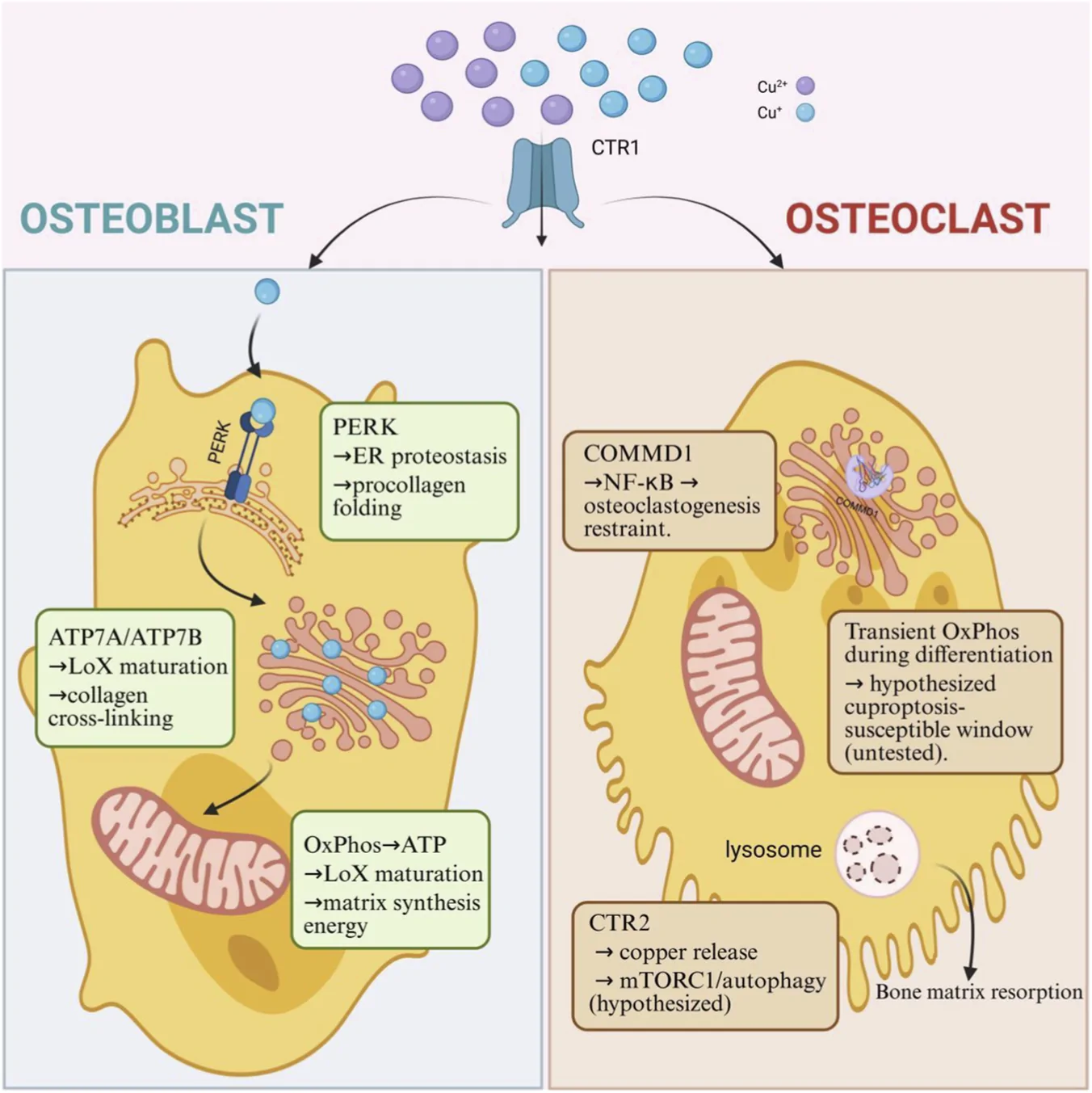
Organelle-level copper handling diverges between osteoblasts and osteoclasts.

Osteoblasts and osteoclasts deploy this network in markedly different ways. Osteoblasts are high-output secretory cells that rely on ER- and TGN-dependent cuproenzyme maturation (LOX for collagen cross-linking) and on mitochondrial oxidative phosphorylation to meet the energetic cost of matrix synthesis. Osteoclasts are terminally differentiated, multinucleated, and highly acidified cells; their resorptive function hinges on lysosomal biogenesis and vesicular trafficking, and their RANKL/NF-κB-driven differentiation is modulated by the copper-dependent regulator copper metabolism MURR1 domain-containing protein 1 (COMMD1). Consequently, identical shifts in whole-cell or systemic copper status can produce divergent, even opposing, effects in the two cell types-a recurring working hypothesis, albeit one not yet rigorously tested by direct side-by-side comparisons under matched copper conditions. Figure 1 provides an integrated schematic of copper trafficking across these five organelles in both osteoblasts and osteoclasts.

### 2.2 Copper uptake

**FIGURE 2:**
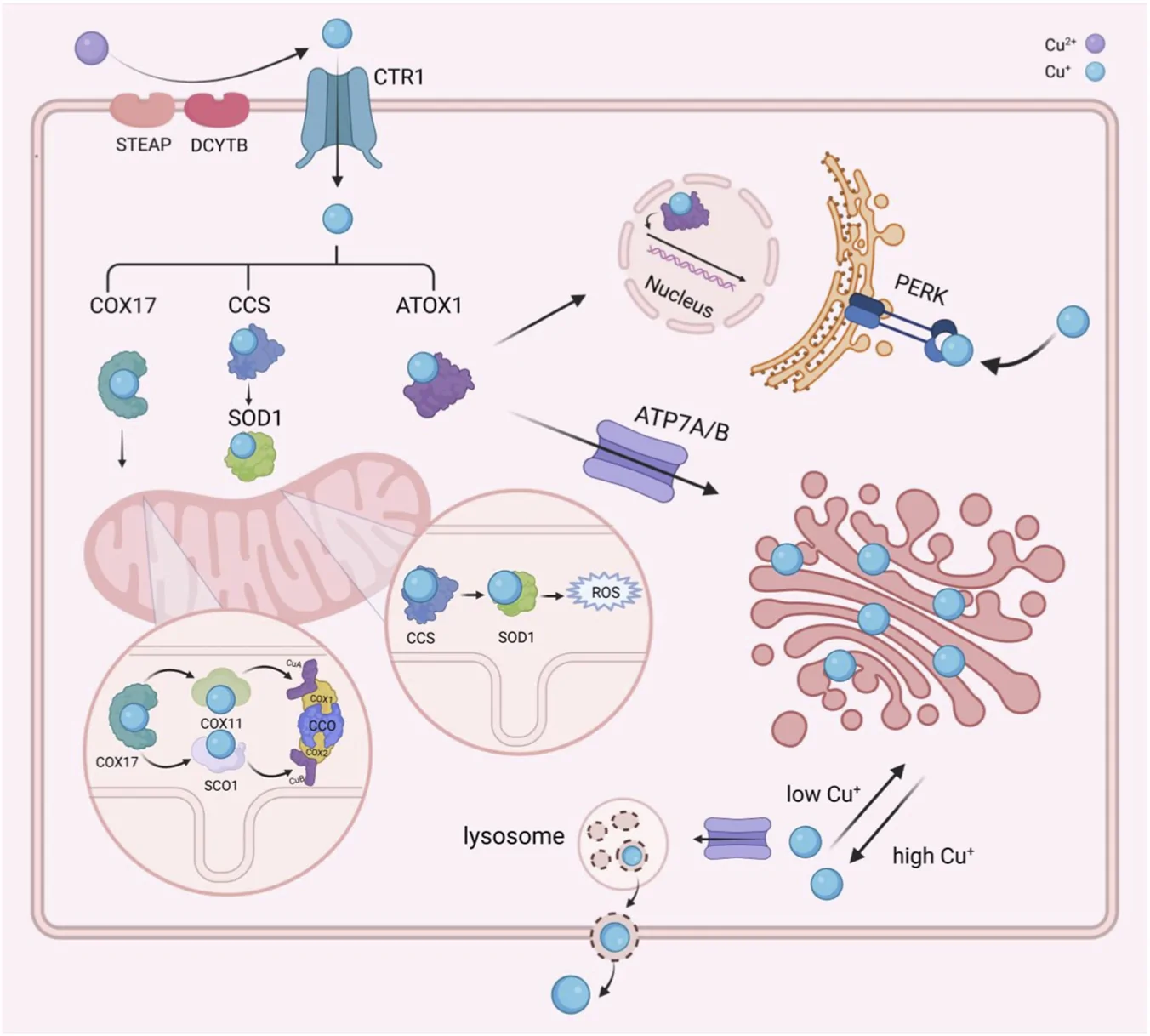
Copper uptake, intracellular distribution, and export at the organelle level.

Copper is mostly present in the oxidised form of Cu2+ in the diet, and ingested Cu2+ is converted to Cu^+^ by metalloreductases through six-transmembrane epithelial antigen of the prostate (STEAP) and duodenal cytochrome b (DCYTB), which promotes cellular acquisition of copper (Ohgami et al., 2006). CTR1 plays an important role in the uptake of Cu+ by mammalian cells (Puig and Thiele, 2002). CTR1 is a transmembrane protein that forms a trimeric transport channel that forms an osmotic pathway for Cu+ through the plasma membrane (Maryon et al., 2013). Copper levels regulate CTR1 expression. Low copper levels upregulate CTR1 expression to increase copper uptake, while high copper levels downregulate CTR1 expression. Therefore, copper homeostasis can be maintained through the control of copper uptake by CTR1 (Kuo et al., 2006; Liang et al., 2012). Figure 2 summarises copper uptake, intracellular distribution, and export.

Cellular copper uptake is governed primarily by intracellular copper status rather than by the abundant circulating pool. Intracellular copper deficiency upregulates CTR1 transcription and membrane localization, whereas sufficiency downregulates CTR1 (Kuo et al., 2006; Liang et al., 2012). In healthy adults, serum copper is maintained at ∼11–22 μmol/L (70–140 μg/dL), overwhelmingly bound to ceruloplasmin (Osredkar and Sustar, 2011). In sharp contrast, free cytosolic copper is virtually undetectable: steady-state estimates suggest fewer than one free copper ion per cell because incoming copper is immediately sequestered by chaperones (ATOX1, CCS, COX17) and low-molecular-weight ligands (Rae et al., 1999). Cells therefore do not sense “copper concentration” in the conventional sense; they sense shifts in copper flux and distribution among chaperone-bound pools. This distinction is essential when interpreting *in vitro* experiments that often apply micromolar concentrations of free copper salts—conditions that do not occur *in vivo*. A central translational bottleneck, echoed throughout this review, originates from this gap: current pharmacological and nutritional strategies modulate bulk, whole-body, or whole-cell copper levels, whereas the organelle-specific mechanisms described below show that therapeutic benefit and toxicity are dictated by subcellular copper distribution, not total cellular copper content. Overcoming this bottleneck will require organelle-targeted delivery technologies that can direct copper to, or withhold it from, specific compartments (e.g., mitochondria versus the trans-Golgi network) instead of uniformly raising or lowering cytosolic copper.

### 2.3 Intracellular copper distribution

After entering the cell, copper first binds to copper molecular chaperones, such as antioxidant protein 1 (ATOX1), cytochrome C oxidase copperchaperone 17 (COX17), copper chaperone for superoxide dismutase (CCS), etc., and are then transported to intracellular compartments, such as mitochondria, the trans-Golgi network (TGN), and the nucleus.

#### 2.3.1 Role of copper molecular chaperones in mitochondria

Mitochondria are central hubs of cellular energy metabolism and regulate essential processes such as redox balance, signal transduction, and apoptosis (Chakrabarty and Chandel, 2021). The electron transport chain (ETC), located on the inner mitochondrial membrane (IMM), generates the proton motive force (PMF) required for oxidative phosphorylation (Ruiz et al., 2021). Copper is an essential mitochondrial cofactor. It is stored in mitochondrial copper ligand pools and is incorporated into cytochrome c oxidase (COX), where it supports electron transfer and ETC activity. Accordingly, mitochondrial copper influences bone formation by regulating oxidative phosphorylation, reactive oxygen species (ROS), and metabolic reprogramming. CCS and SOD1 are localised in the cytoplasmic and mitochondrial intermembrane space (IMS), where they detoxify mitochondria-derived superoxide (Okado-Matsumoto and Fridovich, 2001; Sturtz et al., 2001). CCS is a soluble cytoplasmic copper chaperone protein that transfers copper ions to the copper-binding site of superoxide dismutase 1 (SOD1) (Rosenzweig, 2001). SOD1 is a major antioxidant enzyme that catalyzes the conversion of superoxide radicals to oxygen and hydrogen peroxide, thereby maintaining reactive oxygen species (ROS) homeostasis and protecting cells from oxidative stress damage (Dong et al., 2016). This function has been demonstrated in SOD1 knockout mice, in which a deficiency of SOD1 is associated with oxidative damage, leading first to hepatocellular damage and ultimately to hepatocellular carcinoma (Elchuri et al., 2005).

Copper is involved in the synthesis of COX in mitochondria. In humans, COX contains two core subunits, COX1 and COX2 (cytochrome c oxidase subunit 1/2), and the enzyme has two copper sites in the catalytic core subunit, CuB and CuA sites (Nývltová et al., 2022). COX17 transports copper from the cytoplasm to the mitochondrial membrane compartment (Prohaska, 2008), where it is subsequently transferred to the COX2 subunit via the synthesis of cytochrome C oxidase 1/2 (SCO1/2) or to the COX1 subunit via COX11 and thus participates in electron transfer and oxidative phosphorylation in the respiratory chain (Horng et al., 2004). In addition, COX17 acts as a channel for copper ions to enter the mitochondria within the mitochondrial intermembrane space (IMS) (Horng et al., 2004). Thus, COX17 is essential for proper COX assembly, and COX17 mutations can further reduce COX activity, leading to mitochondrial dysfunction and oxidative stress (Takahashi et al., 2002).

#### 2.3.2 Role of copper molecular chaperones within the trans-Golgi network

ATOX1 delivers copper to ATP7A and ATP7B in the TGN (Lutsenko et al., 2007). ATP7A and ATP7B then transport copper into the TGN lumen, where copper is incorporated into secreted cuproenzymes, including lysyl oxidase, tyrosinase, and ceruloplasmin (Lutsenko et al., 2007). ATP7A is expressed in most organs or tissues, whereas ATP7B is expressed mainly in the liver (Lutsenko et al., 2007). ATP7B is also expressed in intestinal epithelial cells, and is mainly responsible for the storage of copper in vesicles in the cell (Pierson et al., 2018). When excess copper enters the hepatocyte, ATP7B is translocated from the TGN to the lysosome, allowing copper excretion into bile through lysosomal exocytosis (Polishchuk et al., 2014). In addition to its cytosolic chaperone function, ATOX1 can translocate to the nucleus, where it acts as a copper-dependent transcription factor (Itoh et al., 2008).

#### 2.3.3 Copper-ER axis: proteostasis control in osteoblast secretion

Traditionally overlooked in copper trafficking discourse, the endoplasmic reticulum (ER) warrants inclusion in organelle-level analyses of copper-dependent bone metabolism, supported by two pivotal lines of evidence.

First, osteoblasts, among the body’s most secretory cell types, impose an inherent proteostatic burden on the ER by synthesizing and folding substantial amounts of type I procollagen—the primary organic bone matrix constituent—and LOX itself, which requires secretion, glycosylation, and proteolytic processing to activate copper-dependent catalysis. Disruptions to ER folding capacity (e.g., misfolded procollagen accumulation) trigger the unfolded protein response (UPR), suppressing osteoblast differentiation and matrix mineralization if prolonged or excessive. Second, more directly linking copper biology, a recent study revealed that PERK (protein kinase R-like ER kinase), an ER-resident UPR sensor, directly binds copper at specific coordinating residues, a requirement for PERK kinase activity and cellular resilience to ER stress (Bond Newton et al., 2025). This landmark finding establishes a direct molecular tie between intracellular copper availability and ER stress management: cells with impaired copper handling may inadequately mount adaptive UPR responses, while ER-stressed cells may modulate copper demand and redistribute it to the ER. Though unverified in osteoblasts/osteoclasts, this mechanism plausibly explains how both copper deficiency and overload impair osteoblast matrix secretion: deficient cells lack sufficient PERK activity to cope with collagen folding demands intrinsic to osteogenesis, while overload converges on copper-dependent and oxidative-stress pathways to exacerbate ER dysfunction. Notably, this section is framed as an emerging, mechanistically plausible hypothesis rather than an established bone biology pathway, pending direct osteoblast/osteoclast evidence. Identifying ER-copper crosstalk in bone cells is prioritized for future organelle-targeted research.

#### 2.3.4 Lysosomes and endosomes: a copper buffering compartment with cell-type-specific functions in osteoclast resorption

The lysosomal/endosomal system constitutes a second, functionally distinct copper compartment. Human copper transporter 2 (hCTR2) is an oligomeric membrane protein localised to late endosomes and lysosomes. It mobilises copper into the cytosol preferentially under copper-replete or copper-overloaded conditions rather than under basal physiological conditions (van den Berghe et al., 2007). This positions the lysosome as an “overflow” copper reservoir that buffers cytosolic chaperone systems, including ATOX1, CCS, and COX17, against acute fluctuations in copper supply. Consistent with this reservoir function, hepatocytes exposed to excess copper route ATP7B from the TGN to lysosomes, from which copper is expelled into bile via lysosomal exocytosis (Polishchuk et al., 2014).

Beyond copper storage, the lysosome is also the principal signalling platform for mechanistic target of rapamycin complex 1 (mTORC1), a serine/threonine kinase complex that integrates nutrient, energy, and growth-factor signals to control cell growth, protein synthesis, and autophagy; mTORC1 is recruited to and activated at the lysosomal membrane in a manner regulated by lysosomal nutrient and ion content (Rabanal-Ruiz and Korolchuk, 2018). Because lysosomal copper flux can influence local ionic and redox conditions at this membrane, changes in lysosomal copper handling are mechanistically well positioned to modulate mTORC1 activity and, downstream, autophagic flux—although direct evidence for a copper–mTORC1–autophagy axis in bone cells remains unreported, warranting cautious consideration as a plausible hypothesis.

This compartment is of particular relevance to osteoclasts. Unlike osteoblasts, which are principally secretory cells, osteoclasts are terminally differentiated, multinucleated cells whose resorptive function depends on extensive lysosomal biogenesis: osteoclasts assemble a specialised acidified extracellular compartment (the resorption lacuna) via polarised secretion of lysosomal contents, including cathepsin K and tartrate-resistant acid phosphatase, to demineralize and degrade bone matrix. Given that (i) autophagy-related machinery has been shown to support osteoclast ruffled-border formation and secretory function, and (ii) mTORC1 activity is a central negative regulator of autophagy, a copper-responsive lysosome-mTORC1-autophagy axis represents a mechanistically coherent, but still largely untested, candidate pathway linking cellular copper status to osteoclast resorptive capacity. We propose this as a targeted hypothesis for future exploration, as no study has yet experimentally manipulated osteoclast lysosomal copper pools to assess resorptive outcomes.

#### 2.3.5 Peroxisomes: an understudied copper-related compartment

For completeness, we note that peroxisomes harbor a fraction of cellular Cu/Zn superoxide dismutase (SOD1) activity and contribute to reactive oxygen species (ROS) and reactive nitrogen species (RNS) metabolism, raising the possibility of crosstalk with Cu-dependent oxidative pathways discussed above in the mitochondrial ROS section. However, systematic literature searches uncovered no studies directly investigating peroxisomal Cu handling or cell-type-specific Cu transporters in bone cells or other mammalian systems. We therefore identify the peroxisome–Cu–bone axis as an open question, refraining from speculation absent direct evidence. This gap represents a genuine opportunity for future advancement, particularly through the application of subcellular Cu imaging techniques.

#### 2.3.6 Intracellular copper export

In mammals, ATP7A and ATP7B are the principal copper-exporting ATPases that maintain intracellular copper homeostasis. Under physiological copper conditions, both proteins are mainly localised to the TGN, where they transport copper from the cytosol into the TGN lumen for incorporation into secreted cuproenzymes. When intracellular copper rises, ATP7A and ATP7B redistribute from the TGN to vesicular compartments and the plasma membrane, thereby promoting copper efflux; when copper returns to physiological levels, they recycle back to the TGN (Polishchuk et al., 2014). In intestinal epithelial cells, ATP7A mediates basolateral copper export into the portal circulation, allowing copper delivery to the liver, the main organ for systemic copper storage (Linz and Lutsenko, 2007). In hepatocytes, ATP7B mediates copper export into bile, including through vesicular trafficking and lysosomal exocytosis (Polishchuk et al., 2014). In other tissues, such as the placenta and blood-brain barrier, ATP7A contributes to directional copper transport between polarised cells, ensuring copper delivery to the developing fetus and brain (Linz and Lutsenko, 2007).

Mutations in ATP7A and ATP7B cause Menkes disease and Wilson disease, respectively (Horn and Wittung-Stafshede, 2021; Chen et al., 2022). These disorders are modelled in mice by mutations at orthologous loci: Atp7a-mutant “mottled” mice reproduce connective-tissue and skeletal abnormalities of Menkes disease (Lenartowicz et al., 2015), whereas toxic-milk mice and constitutive Atp7b knockout mice reproduce the hepatic copper overload characteristic of Wilson disease. Clinically, ATP7A loss in Menkes disease is associated with reduced lysyl oxidase activity, weakened bone matrix, and low bone mineral density (Kim et al., 2015, p. 201). Radiographic case reports further document wormian bones, rib flaring, metaphyseal spurring, and periosteal reactions in genetically confirmed Menkes disease, consistent with defective LOX-mediated collagen and elastin cross-linking. Conversely, chronic copper overload in Wilson disease is associated with increased fragility fractures and osteoporosis in adult cohorts (Quemeneur et al., 2014; Zhu et al., 2025). These observations at the two extremes of copper homeostasis—deficiency and overload—both converge on impaired bone quality, indicating that bone tissue requires copper to be maintained within a relatively narrow physiological window.

The close homolog hCTR2 localises to late endosomes and lysosomes as an oligomeric membrane protein. Overexpression of hCTR2 stimulates cytosolic release of bioavailable copper selectively under copper-supplemented conditions, typically ≥100 µM extracellular CuSO_4_, approximately 5–10-fold above physiological serum levels, with little effect under basal copper-replete conditions (van den Berghe et al., 2007). This concentration dependence indicates that the lysosomal/endosomal copper pool functions primarily as an overflow reservoir mobilised when cytosolic chaperone capacity is exceeded, rather than as a routine contributor to steady-state copper distribution.

## 3 Copper effects on bone metabolism at the cellular level

### 3.1 Divergent copper demands of osteoblasts and osteoclasts

Osteoblasts and osteoclasts embody fundamentally distinct bioenergetic and secretory programmes, a divergence that dictates how each cell type engages copper at the organelle level. Osteoblasts are professional secretory cells: their continuous synthesis, folding, and export of type I procollagen together with copper-dependent enzymes such as lysyl oxidase (LOX) impose a heavy demand on the ER-to-TGN secretory route. Meeting this secretory burden requires mitochondrial oxidative phosphorylation (OxPhos), which supplies the ATP for matrix production and mineral deposition (Ruiz et al., 2016). Osteoclasts follow a different logic. Osteoclast precursors are glycolytic, but differentiation into mature, resorbing osteoclasts triggers a burst of mitochondrial biogenesis and a shift toward OxPhos—energetically necessary to build and maintain the ruffled border and the acidified resorption lacuna (Park-Min, 2019; Bertels et al., 2024). Once differentiation is largely complete, however, resorptive activity depends more on lysosomal exocytosis than on mitochondrial output.

This metabolic asymmetry carries a direct, testable implication for copper biology. Because OxPhos-dependent cells are markedly more susceptible to copper-induced mitochondrial injury than glycolytic cells, the transient OxPhos-dependent window during osteoclast differentiation could render differentiating osteoclasts uniquely sensitive to copper—a vulnerability that mature osteoclasts and glycolytic osteoblast precursors would not share. This hypothesis arises logically from two independent bodies of work—osteoclast bioenergetics and cuproptosis biology—that have yet to be combined in a single experimental system; no published study has measured cuproptosis susceptibility across osteoclast differentiation stages. We return to this point, and to the complicating influence of the hypoxic bone marrow niche.

A second, better-established asymmetry concerns the organelle-based copper pathways each cell type predominantly relies on. Osteoblast function is disproportionately anchored to the TGN-based ATP7A/ATP7B–LOX axis and, by extension, to ER proteostasis, both of which are secretory in nature. Osteoclast function leans heavily on the lysosomal/endosomal copper pool and the COMMD1–NF-κB axis, pathways that govern acidified vesicular trafficking and RANKL-driven transcription rather than matrix secretion. Recognising this division is essential for interpreting the remainder of this review: interventions that raise or lower whole-cell or systemic copper will not act uniformly on the two cell types, because they are superimposed on two fundamentally different organelle-usage profiles.

### 3.2 Effects of copper homeostasis and copper toxicity on bone metabolism in mitochondria

#### 3.2.1 The biphasic regulation of copper on bone metabolism via mitochondrial ROS homeostasis

**FIGURE 3:**
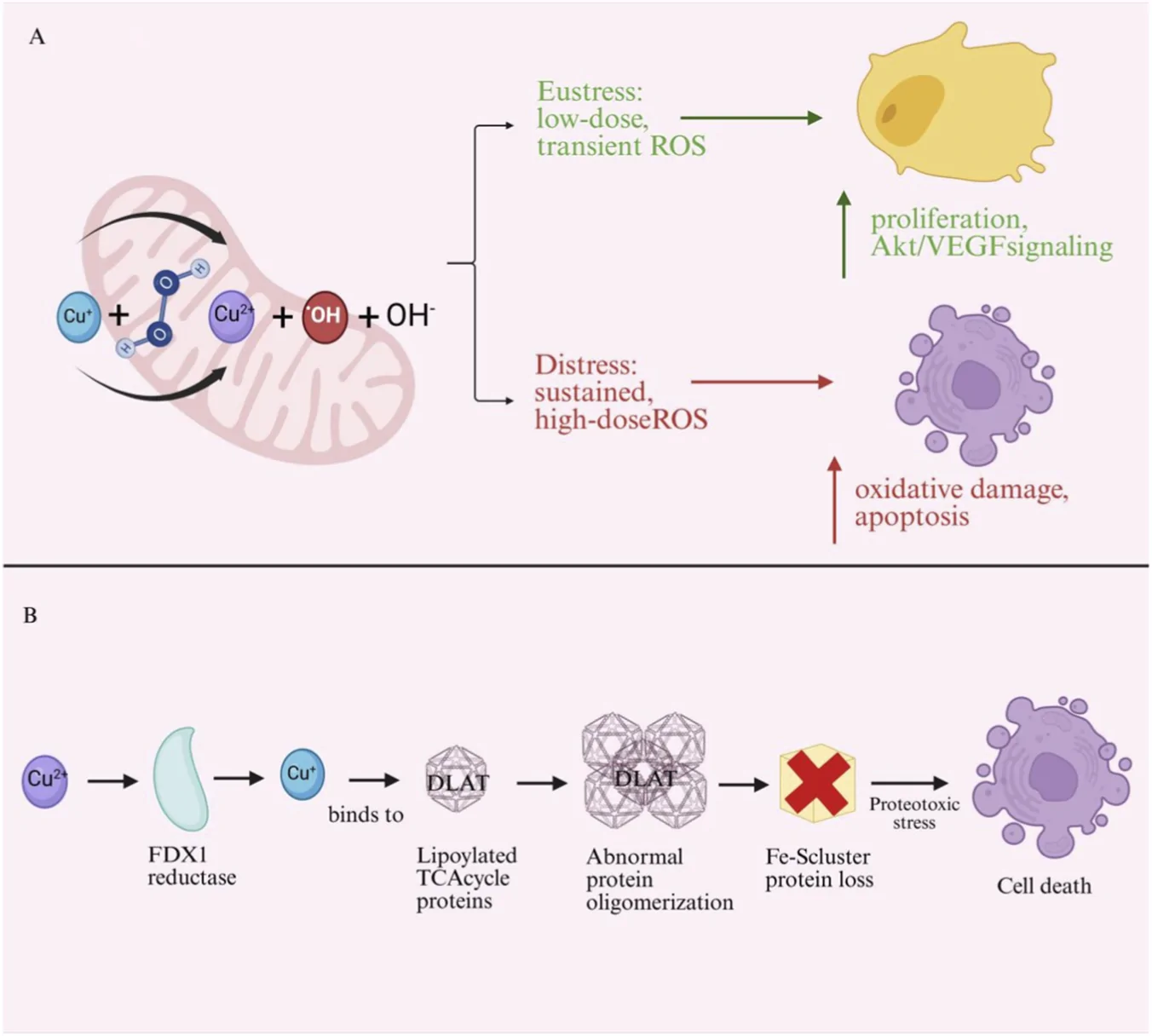
Fenton chemistry, and cuproptosis converge on mitochondrial regulation of bone metabolism. **(A)** Copper-drivenROS:Fenton chemistry. **(B)** Cuproptosis:a distinct mitochondria 1Death pathway.

A moderate elevation of intracellular copper has been demonstrated to accelerate mitochondrial energy metabolism through enhanced oxidative phosphorylation (OxPhos), which can promote cell proliferation and bone formation (Ruiz et al., 2016; Zhang et al., 2024). Rodríguez et al. showed that supplementation with 50 µM copper-histidine (Cu-His) promotes the differentiation of human mesenchymal stem cells (MSCs) into the osteogenic lineage (Rodríguez et al., 2002); it should be noted that this concentration is approximately two- to fourfold above typical serum copper levels (11–22 μmol/L) several orders of magnitude above the vanishingly low free intracellular copper pool (Rae et al., 1999), indicating that this is a supraphysiological, pharmacological intervention rather than a recapitulation of normal physiological copper flux; the translational relevance of this concentration therefore requires cautious interpretation and, ideally, validation at lower, more physiologically representative concentrations. Suh et al. showed that mitochondrial fragmentation promotes the formation of “donut-shaped” mitochondria and enhances mitochondrial-derived vesicle secretion—a process in which fragments of mitochondrial membrane and matrix content are packaged into extracellular vesicles and released from the cell—which in turn accelerates osteoblast osteogenesis in a paracrine manner (Suh et al., 2023); whether this mitochondrial secretory process is itself directly dependent on intracellular copper status has not yet been established and represents an open question. Taken together, these findings suggest that maintenance of mitochondrial copper homeostasis, within a physiologically appropriate range, is important for the process of bone regeneration, although the precise concentration–response relationship *in vivo* remains to be defined. Figure 3 summarises how fenton chemistry and cuproptosis converge on mitochondrial regulation of bone metabolism.

##### 3.2.1.1 Copper as a source of ROS

Copper ions can participate in Fenton-type reactions to generate ROS directly (Sutton and Winterbourn, 1989), in addition to ROS produced by mitochondrial respiration. ROS arise during the sequential, incomplete reduction of molecular oxygen and include the superoxide anion (O_2_•^-^), hydrogen peroxide (H_2_O_2_), and the hydroxyl radical (•OH). Before proceeding, it is important to distinguish “concentration” from “homeostasis”: concentration refers to the measurable amount of ROS or copper present at a given moment, whereas homeostasis refers to the regulated process that maintains that amount within a range compatible with normal cell function.

##### 3.2.1.2 Physiological ROS signalling in osteogenesis

The statement that ROS can “promote” cellular signalling is meaningful only when the concentration and exposure mode are specified. In bone marrow mesenchymal stem cells (BMSCs), low-dose exposure to 50 µM H_2_O_2_ for 12 h enhances proliferation, migration, and survival, partly through upregulation of cyclin D1 and the SDF-1/CXCR4 axis (Guo et al., 2020). This type of low-amplitude signalling is consistent with oxidative eustress, whereas sustained high-level ROS accumulation causes oxidative distress and macromolecular damage (Sies and Jones, 2020). Similarly, continuous low-rate H_2_O_2_ generation by glucose oxidase promotes proliferation and osteoblastic differentiation of human periodontal ligament fibroblasts, as shown by increased alkaline phosphatase activity, mineralisation, and Runx2/Osterix expression; in contrast, a single bolus addition of H_2_O_2_ does not reproduce this effect (Choe et al., 2012). Thus, “appropriate ROS levels” should be understood as low, transient, or continuously generated signals within the eustress range, not as nonspecific ROS elevation.

Beyond these proliferative effects, ROS within the eustress range can support angiogenesis through the Akt pathway, a PI3K-dependent kinase cascade regulating cell survival and metabolism, and through VEGF signalling. FGF-2 has also been reported to maintain a low, pro-survival ROS state in bone marrow stromal and progenitor cells, supporting stromal cell function within the bone marrow microenvironment (Ludin et al., 2014). By contrast, ROS–Notch crosstalk has been characterised mainly in non-skeletal systems, where ROS modulates Notch receptor processing and downstream transcriptional activity. Whether an analogous ROS–Notch mechanism operates during osteogenic differentiation of MSCs remains to be directly demonstrated (Caliceti et al., 2014). Importantly, the mechanisms described in this paragraph refer to ROS signalling in general; they do not require copper as a direct participant.

##### 3.2.1.3 Copper-specific control of ROS via the CCS–SOD1 axis

Having established how ROS itself behaves, we now return to copper’s specific role in regulating ROS abundance. Copper is an essential cofactor for SOD1, which converts superoxide to oxygen and hydrogen peroxide. CTR1-mediated copper uptake and CCS-mediated copper delivery are required for SOD1 maturation and activity (Leary et al., 2009). Copper deficiency can therefore reduce SOD1 activity, shift ROS away from the eustress range, and favour accumulation of superoxide, thereby promoting osteoclast activation and bone resorption. This is consistent with skeletal abnormalities observed in Sod1-deficient mice, including impaired collagen cross-linking and altered bone remodelling (Nojiri et al., 2011) Conversely, copper overload can generate ROS beyond the buffering capacity of CCS–SOD1, disrupting mitochondrial function and cellular metabolism (Bustos et al., 2013; Ruiz et al., 2016). In a controlled therapeutic context, however, copper-containing alloys have been shown to deliberately elevate ROS to promote macrophage recruitment and accelerate bone regeneration (Xu et al., 2020), illustrating that copper-generated ROS can be either harmful or beneficial depending on dose, duration, and cellular context.

##### 3.2.1.4 Consequences of ROS dyshomeostasis for bone cells

When ROS accumulation exceeds the eustress range—whether due to copper overload, mitochondrial dysfunction, or other sources—the resulting oxidative distress inhibits osteogenic capacity, promotes apoptosis, and disrupts osteoblast function (Zhang et al., 2020; Xin et al., 2023). Excess ROS also shapes macrophage recruitment and polarisation, stimulates inflammatory mediator release, and perpetuates inflammatory signalling (Yang et al., 2014; Zhang et al., 2021), while ROS-associated increases in TNF-α further impair osteogenic differentiation of BMSCs (Yang et al., 2023). These downstream consequences apply regardless of whether the initiating ROS surge originates from copper dyshomeostasis or another cellular source.

#### 3.2.2 Cuproptosis: a distinct copper-specific mitochondrial cell death

Physiological copper sustains mitochondrial respiration and antioxidant defence. Overload, however, triggers a mechanistically distinct form of regulated cell death—cuproptosis—that must be clearly separated from ROS-mediated oxidative injury, with which it is often incorrectly conflated in the bone literature.

Tsvetkov et al. (2022) demonstrated that copper ionophores kill cells not primarily through Fenton-type ROS but by driving copper onto lipoylated TCA cycle proteins. The mitochondrial reductase FDX1 reduces relatively inert Cu^2+^ to reactive Cu^+^, which then binds lipoylated components of the pyruvate dehydrogenase complex—most notably dihydrolipoamide S-acetyltransferase (DLAT)—and triggers aberrant disulfide-independent oligomerization. This aggregation is accompanied by destabilisation and loss of iron–sulfur (Fe–S) cluster-containing proteins, causing proteotoxic stress and cell death. A genome-wide CRISPR screen identified ten core pathway genes: seven positive regulators whose loss protects cells (FDX1, LIAS, LIPT1, DLD, DLAT, PDHA1, PDHB) and three negative regulators (MTF1, GLS, CDKN2A) (Tsvetkov et al., 2022). Because cuproptosis requires an intact lipoylation machinery and a functional TCA cycle, respiration-dependent cells are ∼1,000-fold more sensitive than glycolytic cells; glutamine, which feeds the TCA cycle, further tunes susceptibility (Tsvetkov et al., 2022; Li et al., 2023).

This metabolic dependency carries counterintuitive implications for bone. The bone marrow is a hypoxic niche that favours glycolysis over oxidative phosphorylation. Li et al. (2023) therefore proposed that resident osteoblasts, osteoclasts, effector T cells, and macrophages are collectively more glycolytic and thereby relatively protected from cuproptosis—a view that challenges the notion that cuproptosis simply eliminates excess osteoclasts. Hypoxia, however, can simultaneously suppress antioxidant defences and amplify copper toxicity through ROS-dependent routes, meaning its net effect on copper-induced death in bone is not unidirectional. Superimposed on this is a differentiation-stage effect:maturing osteoclasts transiently upregulate oxidative phosphorylation and may therefore pass through a cuproptosis-sensitive window before adopting a lysosome- and glycolysis-dependent mature phenotype. This untested hypothesis predicts that cuproptosis-based interventions, if effective, would best target actively differentiating rather than fully mature osteoclasts.

Clinically, human data complicate the picture further. In 728 postmenopausal women, serum copper was lower, not higher, in osteoporotic individuals and correlated positively with BMD (Li et al., 2023). This is hard to reconcile with a model in which copper excess drives bone loss through osteoblast cuproptosis. Instead, at the population level, copper deficiency—acting through LOX and antioxidant mechanisms—appears to be the dominant axis, while cuproptosis is more relevant to localised high-copper microenvironments than to systemic copper status in ordinary postmenopausal osteoporosis.

Taken together, cuproptosis is a mechanistically well-established death pathway in cancer and other high-turnover systems, yet its relevance, direction of effect, and cell-type selectivity within bone remain genuinely open questions. Small-molecule modulators already available can serve as experimental tools to probe these questions: chlorophyllin, for example, interacts with FDX1 to promote DLAT oligomerization (Xiang et al., 2025), and could be used to compare osteoblast versus osteoclast viability *in vitro*.

### 3.3 Effects of copper homeostasis on bone metabolism in the trans-Golgi network (TGN)

Copper may affect osteoclast differentiation partly through COMMD1, a copper-associated regulator of vesicular trafficking and NF-κB signalling. COMMD1 has been linked to ATP7B stability and copper-loaded vesicle trafficking, suggesting that copper dyshomeostasis may indirectly alter COMMD1-dependent signalling (Liu et al., 2010; Wang et al., 2011).

In osteoclast precursors, COMMD1 is predicted to restrain NF-κB activation and thereby suppress osteoclastogenesis. Mechanistically, COMMD1 promotes ubiquitination and degradation of the NF-κB subunit RelA by interacting with a multimeric ubiquitin ligase complex containing Elongins B and C, Cul2, and SOCS1. Loss of COMMD1 stabilises RelA, increases its nuclear accumulation after TNF stimulation, and enhances κB-responsive transcription (Maine et al., 2007). It should be noted that this ubiquitin-ligase mechanism was originally characterised in non-skeletal human cell lines rather than in osteoclasts directly. Its extension to osteoclast biology is based on the established requirement for NF-κB activation in RANKL-induced osteoclast differentiation, but direct validation of COMMD1-dependent RelA turnover in osteoclast precursors remains lacking.

**FIGURE 4:**
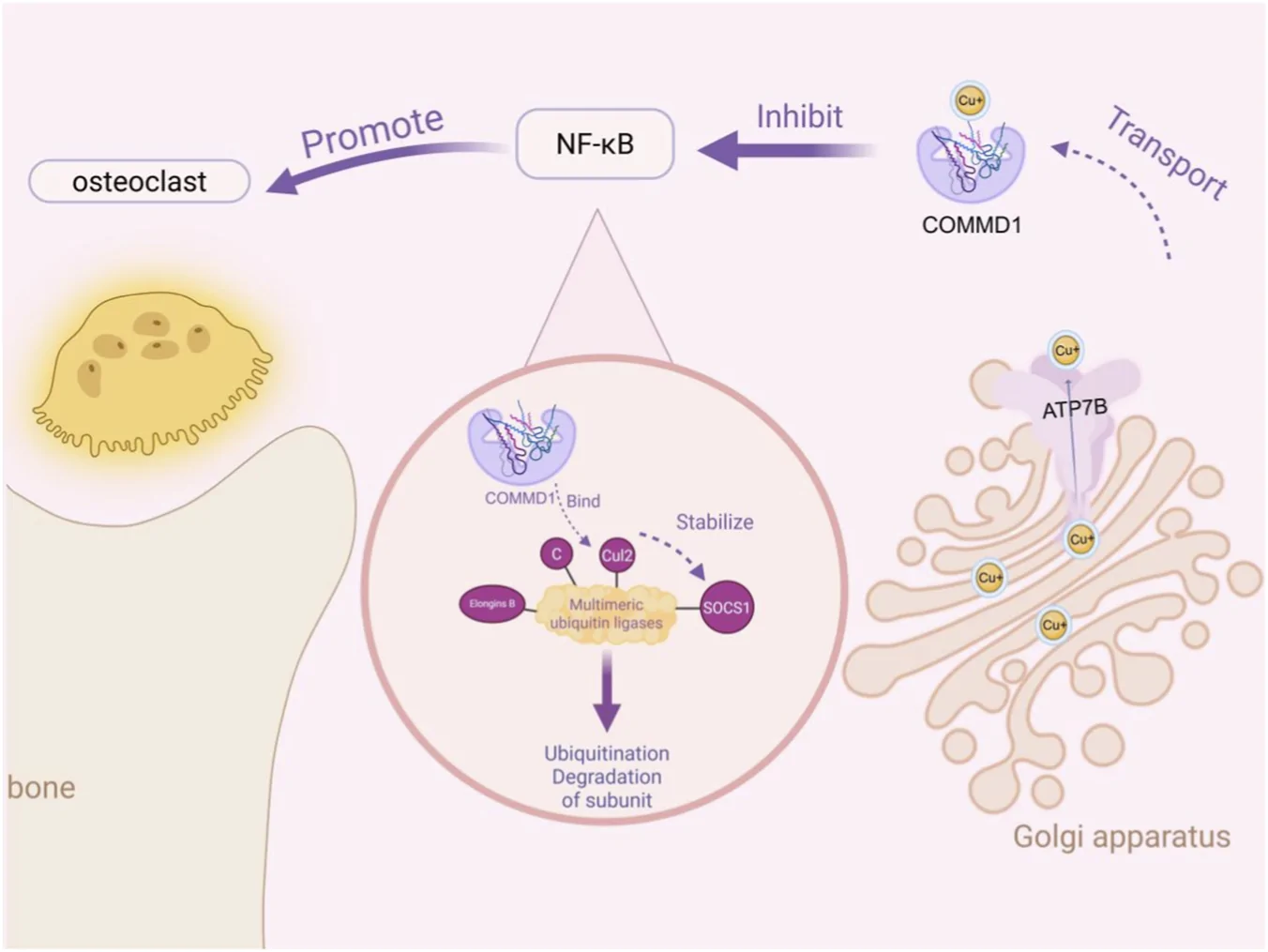
COMMD1 restrains NF-κB signalling to suppress osteoclastogenesis.

Because COMMD1 function is coupled to ATP7B-mediated vesicular copper trafficking, intracellular copper dyshomeostasis is expected to modulate this regulatory axis. Copper deficiency may weaken COMMD1-dependent restraint of NF-κB and thereby favour osteoclastogenesis, whereas copper overload may alter ATP7B/COMMD1 trafficking dynamics in the opposite direction. This remains a prediction derived from copper-trafficking literature rather than a directly tested finding in osteoclasts. Figure 4 illustrates the proposed inhibitory effect of COMMD1 on NF-κB-driven osteoclastogenesis.

### 3.4 Effects of copper homeostasis on bone metabolism in the nucleus

Unlike the COMMD1–NF-κB axis described above, the link between copper status and LOX function is direct rather than inferred: Cu^2+^ serves as an obligatory catalytic cofactor at the LOX active site, meaning that copper deficiency reduces LOX enzymatic activity independently of any change in LOX protein expression. This provides a direct mechanistic route by which systemic or intracellular copper dyshomeostasis translates into impaired collagen cross-linking and the downstream transcriptional effects on RANKL described below.

Copper acts as a catalytic cofactor in redox chemistry and is involved in regulating the activity of various enzymes, and LOX, a secreted copper-dependent amine oxidase, modifies the extracellular matrix of bone (Smith-Mungo and Kagan, 1998). Extracellular matrix-processed LOX can re-enter the cytoplasm to bind to the cytoskeleton and localize in the nucleus of different cell types (Laczko and Csiszar, 2020).

First, LOX can mediate VEGF-induced *in vitro* angiogenesis and osteogenic differentiation of human dental pulp cells. Exogenous VEGF significantly upregulates LOX mRNA and activity while increasing angiogenic mRNA and capillary formation. In contrast, LOX gene silencing and inhibition of LOX activity by BAPN reduced the angiogenic effects of VEGF. Under these conditions, LOX silencing and BAPN inhibited VEGF-induced phosphorylation of AKT, ERK, JNK, and p38, as well as activation of NF-κB (Bae et al., 2018).

Second, active LOX is intimately involved in the mechanisms of cellular fibrosis, particularly in wounds characterized by cycles of injury and repair. During fibrosis, transforming growth factor-β1 (TGF-β1)/Smad3 signaling drives significant upregulation of LOX mRNA, protein levels, and activity in osteoblasts. LOX and type I A1 and A2 collagen genes (COL1A1/A2) have TGF β-responsive promoter elements, which ensures their co-regulated expression (Hong and Trackman, 2002). In bone growth, SMAD4 is a core component of the TGF-β/BMP pathway that controls LOX. Mice lacking Smad4 exhibit a combination of phenotypic features typical of osteogenesis imperfecta, clavicular dysplasia, and Wnt-deficiency syndrome.

**FIGURE 5:**
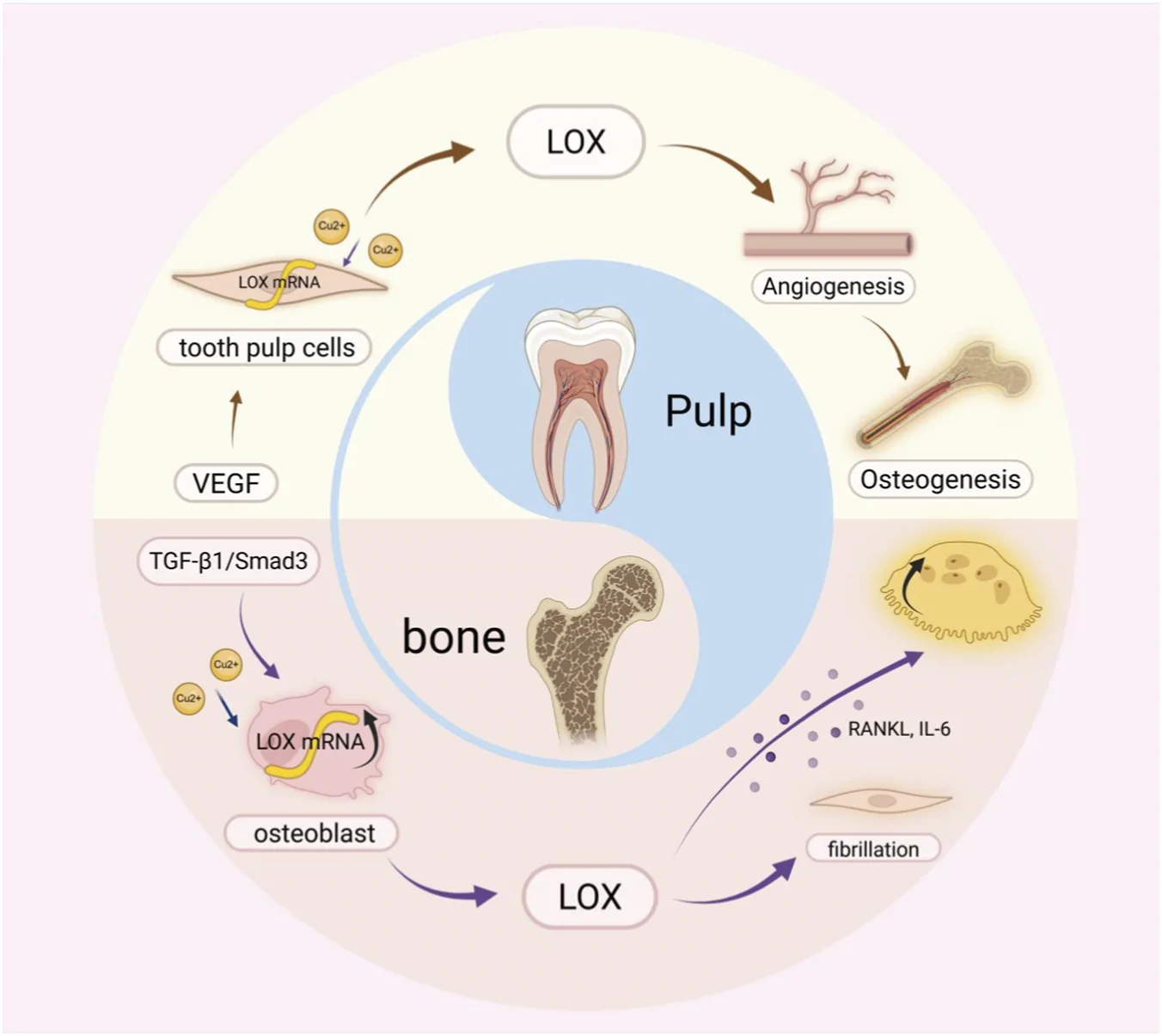
Copper-dependent lysyl oxidase (LOX) bridges osteoblast matrix formation and osteoclast differentiation.

LOX increases the expression of receptor activator of nuclear factor-κB ligand (RANKL) in osteoblasts, which promotes osteoclastogenesis (Tsukasaki et al., 2017). Tumor-secreted LOX and IL-6 synergize to promote RANKL-induced differentiated osteoclastogenesis (Reynaud et al., 2017). LOX overexpression induces the massive production of IL-6, which has the apparent function of promoting bone resorption in osteoclasts. In addition, LOX promotes greater nuclear localization of nuclear factor-activated T cells 1 (NFATc1), a major regulator of osteoclastogenesis. Figure 5 shows how copper-dependent lysyl oxidase (LOX) bridges osteoblast matrix formation and osteoclast differentiation.

### 3.5 Challenges and future directions

The organelle-level mechanisms traced in this review vary widely in how firmly they are established. Some, such as TGN-based maturation of lysyl oxidase, build on decades of enzymology. Others, such as a lysosomal copper–mTORC1–autophagy axis in osteoclasts, are plausible extrapolations from non-skeletal systems that still await direct testing in bone cells. Any translational agenda based on this literature must acknowledge this unevenness rather than smooth it over. Accordingly, we link each direction proposed below to the specific mechanism that motivates it and distinguish ideas ready for pharmacological development from hypotheses that first require direct validation. The following paragraphs lay out this mapping.

Of the three directions, TGN-targeted intervention rests on the firmest ground. Section 2.1 established that LOX must be secreted, glycosylated, and copper-loaded via the ATP7A/ATP7B pathway before it can catalyse the collagen and elastin cross-linking that gives bone matrix its tensile strength, and Section 2.3.2 detailed how these two ATPases are trafficked between the TGN and the plasma or vesicular membrane in response to cytosolic copper levels. Small molecules or oligopeptides designed to stabilise ATP7A/ATP7B in their copper-exporting TGN conformation or to enhance ATOX1-mediated copper delivery to these transporters could in principle increase the pool of properly metalated, catalytically active LOX without raising the whole-cell copper burden—an important distinction, since Section 3.2.2 shows that indiscriminately raising cytosolic copper risks tipping cells toward cuproptosis rather than simply improving enzyme function. Targeted delivery to the TGN, rather than systemic copper loading, is therefore not merely a matter of pharmacological elegance but a direct corollary of the toxicity considerations raised elsewhere in this review.

Among these three directions, mitochondria-targeted copper delivery is the most conceptually ambitious and demands the greatest caution in its presentation. Its appeal is clear: osteoblasts may benefit from moderate mitochondrial copper, whereas cuproptosis offers a mechanistically distinct route for eliminating unwanted cells. A carrier that delivers copper differentially to these two populations could, in theory, simultaneously promote bone formation and suppress excessive resorption. However, this appeal should not be mistaken for an established strategy. Osteoclast bioenergetics depend on the differentiation stage, so any cuproptosis-based selectivity would most plausibly act within a narrow window of active differentiation, not throughout the osteoclast lifespan. Moreover, the hypoxic bone marrow niche favours glycolysis and may blunt cuproptosis sensitivity precisely in the cells this strategy needs to target. Before this direction can reasonably proceed to carrier design, the field needs basic comparative data: under matched, physiologically relevant copper exposures, does cuproptosis susceptibility actually differ between osteoblasts and osteoclasts, and between differentiating and mature osteoclasts? Existing pathway-selective tool compounds, such as the FDX1-interacting agent chlorophyllin (Xiang et al., 2025), provide an accessible starting point for generating such *in vitro* data before any material or delivery system is developed.

The lysosomal/endosomal direction is one step further from clinical relevance than the preceding two, largely because the bone-specific evidence remains thin. CTR2 mobilises copper from the lysosome under copper-replete conditions, and that mTORC1 is activated at the lysosomal membrane, but neither observation has been tested in osteoclasts. The *a priori* rationale is nonetheless strong: lysosomal biogenesis is central to ruffled-border formation and matrix degradation, making osteoclasts a cell type where copper-sensitive lysosomal signalling could well matter. At this stage, the most productive use of this direction is not to propose a lysosome-targeted drug candidate but to drive a more basic experiment—directly measuring how manipulated lysosomal copper (for example, via CTR2 overexpression or knockdown) alters mTORC1 activity, autophagic flux, and resorptive capacity in cultured osteoclasts.

Taken together, these three directions sit at markedly different points on the path from mechanism to therapy. TGN-targeted enhancement of LOX maturation rests on enzymology mature enough to follow a conventional phased pathway: *in vitro* organelle targeting and mechanistic validation, then efficacy and toxicology testing across osteoporosis animal models, then early-phase clinical trials prioritising safety and biomarker endpoints. Mitochondria-targeted and lysosome-targeted strategies are not yet ready for that trajectory. Both draw on mechanisms—cuproptosis and lysosomal copper–mTORC1 coupling, respectively—that are well established in other cellular contexts but have not been directly tested in osteoblasts or osteoclasts. At this stage, both would gain far more from basic comparative experiments than from carrier or delivery-system design. We see this unevenness not as a weakness but as an honest map of where the field stands: one direction has a clear next step toward translation, and two have a clear next step toward the evidence translation requires.

## 4 Discussion

Copper’s relationship to bone is not a simple dose-response story. At either extreme of copper homeostasis—ATP7A loss in Menkes disease, ATP7B loss in Wilson disease—bone quality suffers. Bone tissue evidently needs copper held within a narrow window, and what that copper does once inside the cell depends on which organelle handles it and which cell type is doing the handling. TGN-mediated delivery to lysyl oxidase serves the secretory, matrix-building work of osteoblasts; lysosomal copper handling and the COMMD1–NF-κB axis intersect instead with the resorptive machinery of osteoclasts.

Cuproptosis adds a genuinely new axis to this picture, distinct from copper’s older role in oxidative stress. But the mechanism itself—FDX1-driven aggregation of lipoylated TCA-cycle proteins—is not the same as “cuproptosis selectively kills osteoclasts.” That second claim is still unproven, and the hypoxic bone marrow niche, by favouring glycolysis, may work against it. Any therapeutic reasoning built on this pathway needs to keep these two claims separate rather than treating the first as evidence for the second.

Extending the organelle framework to the ER and the lysosomal/endosomal system—both largely absent from earlier reviews of copper and bone—surfaces two candidate links worth testing directly: copper-dependent PERK activity in ER proteostasis, relevant to osteoblast secretory capacity, and CTR2/mTORC1 coupling at the lysosomal membrane, relevant to osteoclast resorption. Neither has been examined in bone cells; both follow logically from what is already known in other systems.

None of this yet forms a complete map, but it points to where the next experiments should go: measuring cuproptosis susceptibility in osteoblasts versus osteoclasts under matched, physiological copper exposure; testing copper–PERK and copper–mTORC1 signalling directly in bone cells; and continuing to refine TGN-targeted enhancement of lysyl oxidase maturation, which remains the most mechanistically mature route toward translation among the strategies discussed here. The broader lesson is the same across all three: copper’s effect on bone is decided at the level of the organelle and the cell type, not at the level of whole-cell or systemic copper status—and future interventions should be designed accordingly.

## References

[B1] Bae W.-J. Yi J.-K. Park J. Kang S.-K. Jang J.-H. Kim E.-C. (2018). Lysyl oxidase-mediated VEGF-induced differentiation and angiogenesis in human dental pulp cells. Int. Endod. J. 51, 335–346. 10.1111/iej.12796 28568134

[B2] Bertels J. C. He G. Long F. (2024). Metabolic reprogramming in skeletal cell differentiation. Bone Res. 12, 57. 10.1038/s41413-024-00374-0 39394187 PMC11470040

[B3] Bond Newton S. E. Shi X. Beratan N. R. Perhacs J. Arya J. K. Bond M. K. (2025). ER stress tolerance is regulated by copper-dependent PERK kinase activity. Cell Rep. 44, 116318. 10.1016/j.celrep.2025.116318 41014556 PMC12599989

[B4] Bustos R. I. Jensen E. L. Ruiz L. M. Rivera S. Ruiz S. Simon F. (2013). Copper deficiency alters cell bioenergetics and induces mitochondrial fusion through up-regulation of MFN2 and OPA1 in erythropoietic cells. Biochem. Biophys. Res. Commun. 437, 426–432. 10.1016/j.bbrc.2013.06.095 23831624

[B5] Caliceti C. Nigro P. Rizzo P. Ferrari R. (2014). ROS, notch, and wnt signaling pathways: crosstalk between three major regulators of cardiovascular biology. Biomed. Res. Int. 2014, 1–8. 10.1155/2014/318714 24689035 PMC3932294

[B6] Chakrabarty R. P. Chandel N. S. (2021). Mitochondria as signaling organelles control mammalian stem cell fate. Cell Stem Cell 28, 394–408. 10.1016/j.stem.2021.02.011 33667360 PMC7944920

[B7] Chen L. Min J. Wang F. (2022). Copper homeostasis and cuproptosis in health and disease. Signal Transduct. Target. Ther. 7, 378. 10.1038/s41392-022-01229-y 36414625 PMC9681860

[B8] Choe Y. Yu J. Son Y. Park S. Kim J. Shi X. (2012). Continuously generated H_2_ O_2_ stimulates the proliferation and osteoblastic differentiation of human periodontal ligament fibroblasts. J. Cell. Biochem. 113, 1426–1436. 10.1002/jcb.24017 22173791 PMC3340504

[B9] Dong X. Zhang Z. Zhao J. Lei J. Chen Y. Li X. (2016). The rational design of specific SOD1 inhibitors *via* copper coordination and their application in ROS signaling research. Chem. Sci. 7, 6251–6262. 10.1039/C6SC01272H 30034766 PMC6024207

[B10] Elchuri S. Oberley T. D. Qi W. Eisenstein R. S. Jackson Roberts L. Van Remmen H. (2005). CuZnSOD deficiency leads to persistent and widespread oxidative damage and hepatocarcinogenesis later in life. Oncogene 24, 367–380. 10.1038/sj.onc.1208207 15531919

[B11] Fan Y. Ni S. Zhang H. (2022). Associations of copper intake with bone mineral density and osteoporosis in adults: data from the national health and nutrition examination survey. Biol. Trace Elem. Res. 200, 2062–2068. 10.1007/s12011-021-02845-5 34283365

[B12] Guo L. Du J. Yuan D. Zhang Y. Zhang S. Zhang H. (2020). Optimal H2O2 preconditioning to improve bone marrow mesenchymal stem cells’ engraftment in wound healing. Stem Cell Res. Ther. 11, 434. 10.1186/s13287-020-01910-5 33032649 PMC7545926

[B13] Hong H.-H. Trackman P. C. (2002). Cytokine regulation of gingival fibroblast lysyl oxidase, collagen, and elastin. J. Periodontol. 73, 145–152. 10.1902/jop.2002.73.2.145 11895278

[B29] Horn N. Wittung-Stafshede P. (2021). ATP7A-Regulated enzyme metalation and trafficking in the Menkes disease puzzle. Biomedicines 9, 391. 10.3390/biomedicines9040391 33917579 PMC8067471

[B14] Horng Y.-C. Cobine P. A. Maxfield A. B. Carr H. S. Winge D. R. (2004). Specific copper transfer from the Cox17 metallochaperone to both Sco1 and Cox11 in the assembly of yeast cytochrome c oxidase. J. Biol. Chem. 279, 35334–35340. 10.1074/jbc.M404747200 15199057

[B15] Itoh S. Kim H. W. Nakagawa O. Ozumi K. Lessner S. M. Aoki H. (2008). Novel role of Antioxidant-1 (Atox1) as a copper-dependent transcription factor involved in cell proliferation. J. Biol. Chem. 283, 9157–9167. 10.1074/jbc.M709463200 18245776 PMC2431038

[B16] Kim D. Choi J. Han K.-M. Lee B. H. Choi J.-H. Yoo H.-W. (2015). Impaired osteogenesis in Menkes disease-derived induced pluripotent stem cells. Stem Cell Res. Ther. 6, 160. 10.1186/s13287-015-0147-5 26347346 PMC4562349

[B17] Kuo Y.-M. Gybina A. A. Pyatskowit J. W. Gitschier J. Prohaska J. R. (2006). Copper transport protein (Ctr1) levels in mice are tissue specific and dependent on copper status. J. Nutr. 136, 21–26. 10.1093/jn/136.1.21 16365053 PMC2718570

[B18] Laczko R. Csiszar K. (2020). Lysyl oxidase (LOX): functional contributions to signaling pathways. Biomolecules 10, 1093. 10.3390/biom10081093 32708046 PMC7465975

[B19] Leary S. C. Winge D. R. Cobine P. A. (2009). “Pulling the plug” on cellular copper: the role of mitochondria in copper export. Biochim. Biophys. Acta 1793, 146–153. 10.1016/j.bbamcr.2008.05.002 18522804 PMC4021392

[B20] Lenartowicz M. Krzeptowski W. Lipiński P. Grzmil P. Starzyński R. Pierzchała O. (2015). Mottled mice and non-mammalian models of Menkes disease. Front. Mol. Neurosci. 8. 10.3389/fnmol.2015.00072 26732058 PMC4684000

[B21] Li D. Gao Z. Li Q. Liu X. Liu H. (2023). Cuproptosis-a potential target for the treatment of osteoporosis. Front. Endocrinol. 14, 1135181. 10.3389/fendo.2023.1135181 37214253 PMC10196240

[B22] Liang Z. D. Tsai W.-B. Lee M.-Y. Savaraj N. Kuo M. T. (2012). Specificity protein 1 (Sp1) oscillation is involved in copper homeostasis maintenance by regulating human high-affinity copper transporter 1 expression. Mol. Pharmacol. 81, 455–464. 10.1124/mol.111.076422 22172574 PMC3286298

[B23] Linz R. Lutsenko S. (2007). Copper-transporting ATPases ATP7A and ATP7B: cousins, not twins. J. Bioenerg. Biomembr. 39, 403–407. 10.1007/s10863-007-9101-2 18000748

[B68] Liu Y. Pilankatta R. Hatori Y. Lewis D. Inesi G. (2010). Comparative features of copper ATPases ATP7A and ATP7B heterologously expressed in COS-1 cells. Biochemistry 49, 10006–10012. 10.1021/bi101423j 20964302 PMC2982669

[B24] Ludin A. Gur-Cohen S. Golan K. Kaufmann K. B. Itkin T. Medaglia C. (2014). Reactive oxygen species regulate hematopoietic stem cell self-renewal, migration and development, As well As their bone marrow microenvironment. Antioxid. Redox Signal. 21, 1605–1619. 10.1089/ars.2014.5941 24762207 PMC4175025

[B25] Lutsenko S. Barnes N. L. Bartee M. Y. Dmitriev O. Y. (2007). Function and regulation of human copper-transporting ATPases. Physiol. Rev. 87, 1011–1046. 10.1152/physrev.00004.2006 17615395

[B26] Maine G. N. Mao X. Komarck C. M. Burstein E. (2007). COMMD1 promotes the ubiquitination of NF-kappaB subunits through a cullin-containing ubiquitin ligase. EMBO J. 26, 436–447. 10.1038/sj.emboj.7601489 17183367 PMC1783443

[B27] Maryon E. B. Molloy S. A. Ivy K. Yu H. Kaplan J. H. (2013). Rate and regulation of copper transport by human copper transporter 1 (hCTR1). J. Biol. Chem. 288, 18035–18046. 10.1074/jbc.M112.442426 23658018 PMC3689948

[B28] Morisawa A. Okui T. Shimo T. Ibaragi S. Okusha Y. Ono M. (2018). Ammonium tetrathiomolybdate enhances the antitumor effects of cetuximab *via* the suppression of osteoclastogenesis in head and neck squamous carcinoma. Int. J. Oncol. 52, 989–999. 10.3892/ijo.2018.4242 29328370

[B30] Nojiri H. Saita Y. Morikawa D. Kobayashi K. Tsuda C. Miyazaki T. (2011). Cytoplasmic superoxide causes bone fragility owing to low-turnover osteoporosis and impaired collagen cross-linking. J. Bone Min. Res. Off. J. Am. Soc. Bone Min. Res. 26, 2682–2694. 10.1002/jbmr.489 22025246

[B31] Nývltová E. Dietz J. V. Seravalli J. Khalimonchuk O. Barrientos A. (2022). Coordination of metal center biogenesis in human cytochrome c oxidase. Nat. Commun. 13, 3615. 10.1038/s41467-022-31413-1 35750769 PMC9232578

[B32] Ohgami R. S. Campagna D. R. McDonald A. Fleming M. D. (2006). The steap proteins are metalloreductases. Blood 108, 1388–1394. 10.1182/blood-2006-02-003681 16609065 PMC1785011

[B33] Okado-Matsumoto A. Fridovich I. (2001). Subcellular distribution of superoxide dismutases (SOD) in rat liver. J. Biol. Chem. 276, 38388–38393. 10.1074/jbc.M105395200 11507097

[B34] Osredkar J. Sustar N. (2011). Copper and zinc, biological role and significance of copper/zinc imbalance. J. Clin. Toxicol. s3. 10.4172/2161-0495.S3-001

[B35] Park-Min K.-H. (2019). Metabolic reprogramming in osteoclasts. Semin. Immunopathol. 41, 565–572. 10.1007/s00281-019-00757-0 31552471 PMC7671717

[B36] Pierson H. Muchenditsi A. Kim B.-E. Ralle M. Zachos N. Huster D. (2018). The function of ATPase copper transporter ATP7B in intestine. Gastroenterology 154, 168–180.e5. 10.1053/j.gastro.2017.09.019 28958857 PMC5848507

[B37] Polishchuk E. V. Concilli M. Iacobacci S. Chesi G. Pastore N. Piccolo P. (2014). Wilson disease protein ATP7B utilizes lysosomal exocytosis to maintain copper homeostasis. Dev. Cell 29, 686–700. 10.1016/j.devcel.2014.04.033 24909901 PMC4070386

[B38] Prohaska J. R. (2008). Role of copper transporters in copper homeostasis. Am. J. Clin. Nutr. 88, 826S–829S. 10.1093/ajcn/88.3.826S 18779302 PMC2799992

[B39] Puig S. Thiele D. J. (2002). Molecular mechanisms of copper uptake and distribution. Curr. Opin. Chem. Biol. 6, 171–180. 10.1016/S1367-5931(02)00298-3 12039001

[B40] Quemeneur A.-S. Trocello J.-M. Ea H.-K. Ostertag A. Leyendecker A. Duclos-Vallée J.-C. (2014). Bone status and fractures in 85 adults with Wilson’s disease. Osteoporos. Int. 25, 2573–2580. 10.1007/s00198-014-2806-2 25027110

[B41] Rabanal-Ruiz Y. Korolchuk V. (2018). mTORC1 and nutrient homeostasis: the central role of the lysosome. Int. J. Mol. Sci. 19, 818. 10.3390/ijms19030818 29534520 PMC5877679

[B42] Rae T. D. Schmidt P. J. Pufahl R. A. Culotta V. C. V. O’Halloran T. (1999). Undetectable intracellular free copper: the requirement of a copper chaperone for superoxide dismutase. Science 284, 805–808. 10.1126/science.284.5415.805 10221913

[B43] Reynaud C. Ferreras L. Di Mauro P. Kan C. Croset M. Bonnelye E. (2017). Lysyl oxidase is a strong determinant of tumor cell colonization in bone. Cancer Res. 77, 268–278. 10.1158/0008-5472.CAN-15-2621 27742687

[B44] Rodríguez J. P. Ríos S. González M. (2002). Modulation of the proliferation and differentiation of human mesenchymal stem cells by copper. J. Cell. Biochem. 85, 92–100. 10.1002/jcb.10111 11891853

[B45] Rosenzweig A. C. (2001). Copper delivery by metallochaperone proteins. Acc. Chem. Res. 34, 119–128. 10.1021/ar000012p 11263870

[B46] Ruiz L. M. Jensen E. L. Rossel Y. Puas G. I. Gonzalez-Ibanez A. M. Bustos R. I. (2016). Non-cytotoxic copper overload boosts mitochondrial energy metabolism to modulate cell proliferation and differentiation in the human erythroleukemic cell line K562. Mitochondrion 29, 18–30. 10.1016/j.mito.2016.04.005 27094959

[B47] Ruiz L. M. Libedinsky A. Elorza A. A. (2021). Role of copper on mitochondrial function and metabolism. Front. Mol. Biosci. 8, 711227. 10.3389/fmolb.2021.711227 34504870 PMC8421569

[B48] Sies H. Jones D. P. (2020). Reactive oxygen species (ROS) as pleiotropic physiological signalling agents. Nat. Rev. Mol. Cell Biol. 21, 363–383. 10.1038/s41580-020-0230-3 32231263

[B49] Smith-Mungo L. I. Kagan H. M. (1998). Lysyl oxidase: properties, regulation and multiple functions in biology. Matrix Biol. J. Int. Soc. Matrix Biol. 16, 387–398. 10.1016/s0945-053x(98)90012-9 9524359

[B50] Sturtz L. A. Diekert K. Jensen L. T. Lill R. Culotta V. C. (2001). A fraction of yeast Cu,Zn-Superoxide dismutase and its metallochaperone, CCS, localize to the intermembrane space of Mitochondria. J. Biol. Chem. 276, 38084–38089. 10.1074/jbc.M105296200 11500508

[B51] Suh J. Kim N.-K. Shim W. Lee S.-H. Kim H.-J. Moon E. (2023). Mitochondrial fragmentation and donut formation enhance mitochondrial secretion to promote osteogenesis. Cell Metab. 35, 345–360.e7. 10.1016/j.cmet.2023.01.003 36754021

[B52] Sutton H. C. Winterbourn C. C. (1989). On the participation of higher oxidation states of iron and copper in Fenton reactions. Free Radic. Biol. Med. 6, 53–60. 10.1016/0891-5849(89)90160-3 2536343

[B53] Takahashi Y. Kako K. Kashiwabara S. Takehara A. Inada Y. Arai H. (2002). Mammalian copper chaperone Cox17p has an essential role in activation of cytochrome *c* oxidase and embryonic development. Mol. Cell. Biol. 22, 7614–7621. 10.1128/MCB.22.21.7614-7621.2002 12370308 PMC135665

[B54] Tsukasaki M. Hamada K. Okamoto K. Nagashima K. Terashima A. Komatsu N. (2017). LOX fails to substitute for RANKL in osteoclastogenesis. J. Bone Min. Res. Off. J. Am. Soc. Bone Min. Res. 32, 434–439. 10.1002/jbmr.2990 27606829

[B55] Tsvetkov P. Coy S. Petrova B. Dreishpoon M. Verma A. Abdusamad M. (2022). Copper induces cell death by targeting lipoylated TCA cycle proteins. Science 375, 1254–1261. 10.1126/science.abf0529 35298263 PMC9273333

[B56] van den Berghe P. V. E. Folmer D. E. Malingré H. E. M. van Beurden E. Klomp A. E. M. van de Sluis B. (2007). Human copper transporter 2 is localized in late endosomes and lysosomes and facilitates cellular copper uptake. Biochem. J. 407, 49–59. 10.1042/BJ20070705 17617060 PMC2267400

[B69] Wang Y. Hodgkinson V. Zhu S. Weisman G. A. Petris M. J. (2011). Advances in the understanding of mammalian copper transporters. Advances in Nutrition. 2, 129–137. 10.3945/an.110.000273 22332042 PMC3065767

[B57] Wu A.-M. Bisignano C. James S. L. Abady G. G. Abedi A. Abu-Gharbieh E. (2021). Global, regional, and national burden of bone fractures in 204 countries and territories, 1990–2019: a systematic analysis from the global burden of disease study 2019. Lancet Healthy Longev. 2, e580–e592. 10.1016/S2666-7568(21)00172-0 34723233 PMC8547262

[B58] Xiang Z. Mei H. Wang H. Yao X. Rao J. Zhang W. (2025). Cuproptosis and its potential role in musculoskeletal disease. Front. Cell Dev. Biol. 13, 1570131. 10.3389/fcell.2025.1570131 40292330 PMC12022686

[B59] Xin L. Wen Y. Song J. Chen T. Zhai Q. (2023). Bone regeneration strategies based on organelle homeostasis of mesenchymal stem cells. Front. Endocrinol. Lausanne 14, 1151691. 10.3389/fendo.2023.1151691 37033227 PMC10081449

[B60] Xu D. Qian J. Guan X. Ren L. Yang K. Huang X. (2020). Copper-containing alloy as immunoregulatory material in bone regeneration *via* mitochondrial oxidative stress. Front. Bioeng. Biotechnol. 8, 620629. 10.3389/fbioe.2020.620629 33569374 PMC7869892

[B61] Yang W. S. SriRamaratnam R. Welsch M. E. Shimada K. Skouta R. Viswanathan V. S. (2014). Regulation of ferroptotic cancer cell death by GPX4. Cell 156, 317–331. 10.1016/j.cell.2013.12.010 24439385 PMC4076414

[B62] Yang M. Shen Z. Zhang X. Song Z. Zhang Y. Lin Z. (2023). Ferroptosis of macrophages facilitates bone loss in apical periodontitis *via* NRF2/FSP1/ROS pathway. Free Radic. Biol. Med. 208, 334–347. 10.1016/j.freeradbiomed.2023.08.020 37619958

[B63] Zhang Q. Cheng X. Zhang H. Zhang T. Wang Z. Zhang W. (2020). Dissecting molecular mechanisms underlying H(2)O(2)-induced apoptosis of mouse bone marrow mesenchymal stem cell: role of Mst1 inhibition. Stem Cell Res. Ther. 11, 526. 10.1186/s13287-020-02041-7 33298178 PMC7724846

[B64] Zhang B. Yang Y. Yi J. Zhao Z. Ye R. (2021). Hyperglycemia modulates M1/M2 macrophage polarization *via* reactive oxygen species overproduction in ligature-induced periodontitis. J. Periodontal Res. 56, 991–1005. 10.1111/jre.12912 34190354

[B65] Zhang Q. Pan R. L. Wang H. Wang J. J. Lu S. H. Zhang M. (2024). Nanoporous titanium implant surface accelerates osteogenesis *via* the Piezo1/Acetyl-CoA/β-Catenin pathway. Nano Lett. 24, 8257–8267. 10.1021/acs.nanolett.4c01101 38920296 PMC11247543

[B66] Zhu J. Liao Y. Li X. Jia F. Ma X. Qu H. (2024). Brain and the whole-body bone imaging appearances in menkes disease: a case report and literature review. BMC Pediatr. 24, 411. 10.1186/s12887-024-04885-x 38926644 PMC11202368

[B67] Zhu L. Song B. Xu Y. Zhu Y. Hua L. Nian N. (2025). Osteoporosis in Wilson’s disease: a large cohort study highlighting age, sex and skeletal symptoms as key risk factors for clinical surveillance. Orphanet J. Rare Dis. 20, 388. 10.1186/s13023-025-03910-1 40731295 PMC12309045

